# Hydrodynamic lubrication effects in textured PEEK surfaces for friction reduction

**DOI:** 10.1007/s44245-025-00130-6

**Published:** 2025-09-29

**Authors:** Christopher W. Harris, Omid Doustdar, Arvin Taghizadeh Tabrizi, Ali M. Al-Qahtani, Amir M. Hajiyavand, Karl D. Dearn

**Affiliations:** 1 Department of Mechanical Engineering, School of Engineering, University of Birmingham, Birmingham, B15 2TT UK; 2 Department of Chemical Engineering, Toronto Metropolitan University, Toronto, Canada; 3 Mechanical Engineering Department, Jubail Industrial College, 31961 Jubail, Saudi Arabia

**Keywords:** Surface textures, PEEK, Water lubrication, Friction, Bearing

## Abstract

The tribology of dimple-shaped textures is investigated within a reciprocating polymeric sliding couple of Invibio poly-ether-ether-ketone (grade 450G). A novel theoretical model was developed for this aim, describing hydrodynamic pressure and frictional force due to surface modifications. Experiments show reduced friction across all textures compared to un-textured surfaces. The parameters of texture depth (5–20 µm) and diameter (50–200 µm) were isolated to analyse the effect of each independently. The diameter showed good agreement with the model; however, depth showed variation at the extremities of the parameters examined. The experimental friction force captured over a single stroke was compared to the instantaneous theoretical friction force, and good agreement was found in the form of the friction trace within the hydrodynamic region of a reciprocating stroke. The texture parameter combination of 50 µm diameter and 20 µm depth showed the most significant reduction in friction at 48.7%.

**Graphical abstract:**

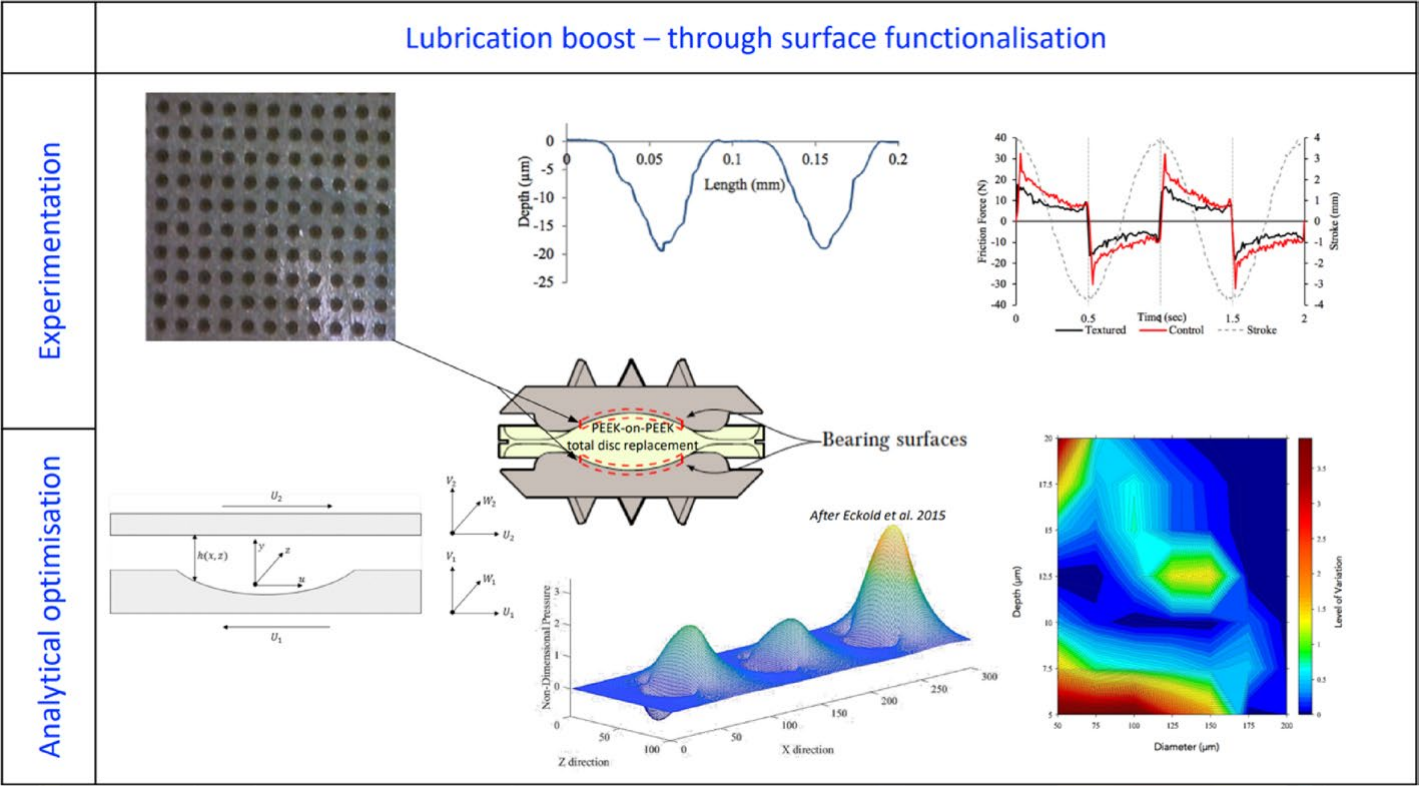

## Introduction

Friction control of articulating prosthetic surfaces is an integral aspect of the mechanical design of medical devices. An imperative factor is preserving the integrity of the surface of a component to enhance the longevity and efficiency of the device's service life. In the field of biomedical engineering, optimizing the performance and longevity of implant materials is of paramount importance. Joint replacements, such as those used in total hip, knee, and disc arthroplasty, are continually being improved to mitigate issues related to wear, friction, and material degradation. Current material combinations for total joint arthroplasty (TJA) tend to be metal-on-metal, polymer-on-metal, or ceramic-on-ceramic. Although such combinations are widely implemented, problems still exist with the durability of such prostheses. Common causes of failure within ultra-high-molecular-weight-polyethylene (UHMWPE)-on-metal combinations include osteolysis, induced by the generation of wear debris that leads to prosthetic loosening [1]. For metal-on-metal prosthetic wear couples, while initially introduced to improve longevity and reduce the amount of wear debris, friction and wear processes produce metal ion accumulations that pose certain health risks [2]. PEEK can be replaced with traditional materials (metals and polymers such as Nylon and PTFE) in diverse applications, particularly where weight, corrosion resistance, and noise reduction are important. Compared to the polymer bearings, PEEK offers better performance in high-temperature and high-load applications. However, PEEK has a higher coefficient of thermal expansion compared to metals, which restricts its application.

Additionally, the in situ generation of metallic wear nanoparticles, especially cobalt and chromium, and the release of trace metal ions into the body from metallic implants can lead to local nanotoxicity effects and contribute to implant failure [3]. Over time, these ions can accumulate to toxic levels, creating significant health risks such as hypersensitivity reactions, pseudotumor formation, and even systemic toxicity, leading to concerns about their long-term safety in patients. Ceramic-on-ceramic sliding couples have shown exemplary promise, attaining low friction results within joint simulator studies. However, ceramic components' brittleness and reduced toughness could lead to component fracture [4]. Also, ceramic-on-ceramic sliding couples have demonstrated low friction behaviour in joint simulator studies [5, 6].

Although not as widely studied, the potential of polymer-on-polymer combinations is of interest within the bio-medical field. All polymer tribo-couples, for example, have been studied in-vitro spine simulator tests for cervical total disc replacement (TDR) [7]. Polymers can provide greater design flexibility regarding the conformability of mating components and the potential for application-specific materials through polymer composite design. Furthermore, the prospect of all polymer implants could reduce stress shielding that induces bone resorption that occurs using harder materials. In other words, polymer composites offer a promising alternative to traditional metallic implants in orthopaedic applications due to their ability to address stress shielding and promote better bone healing. Combining polymer composite technology with nanotechnology can develop polymer nanocomposites with enhanced mechanical and biological properties suitable for biomedical applications [8, 9]. However, the friction and subsequent wear of soft articulating surfaces remain prominent in the polymer device design [10]. Invibio PEEK (poly-ether-ether-ketone, grade 450G) was specifically chosen for this study due to its excellent combination of mechanical, thermal, and tribological properties, which make it highly suitable for engineering applications, particularly in demanding environments such as biomedical implants, aerospace, and automotive components. PEEK exhibits superior wear resistance, low friction coefficients, and high chemical stability, all of which are essential for evaluating the hydrodynamic-lubrication effects and frictional characteristics of textured surfaces in reciprocating sliding. The focus of this research was to assess the tribological performance of textured PEEK surfaces under hydrodynamic lubrication. Although other polymeric materials were considered, PEEK was prioritized due to its wide application in both industrial and biomedical sectors, where minimizing friction and wear is critical. Additionally, PEEK’s ability to retain its properties in a broad range of temperatures and chemical environments makes it a relevant material for real-world scenarios where these factors influence performance [11]. Comparing the tribological behavior of other polymers, such as PTFE (polytetrafluoroethylene), UHMWPE (ultra-high-molecular-weight polyethylene), or nylon, would indeed offer insightful contrasts. For instance, PTFE has an even lower friction coefficient than PEEK but tends to have poorer wear resistance. UHMWPE exhibits excellent wear resistance but lacks the high-temperature stability of PEEK. Nylon is widely used in tribological applications but generally performs sub-optimally in high-temperature or chemically aggressive environments compared to PEEK. Nevertheless, understanding the tribology of such devices is critical as the size and morphology of wear debris generated have been shown to evolve during the life of a device [12]. Nine et al. observed that the wear debris' size, shape, and volume are influenced by the types of joints, the geometry of the bearings, and the type of lubricant used [13].

The tribological behaviour of polymers and their composites is influenced by load, sliding velocity, and temperature, with wear occurring through abrasive, adhesive, and fatigue mechanisms. However, friction-induced wear mechanisms of polymers, such as surface fatigue, culminating in the expulsion of particulates from the bulk material, represent a significant obstacle in the operational longevity of polymeric-based artificial joints. Various methods have been employed to enhance the tribological properties of polymers, including filler incorporation into polymer composites (carbon-filled, cross-linked), gamma irradiation, and surface texturing [14, 15]. For instance, Dougherty et al. [16] reported that microdimple texturing can trap wear debris and promote mixed to full film lubrication, potentially reducing friction by up to 50%. Such progress in polymer technology is essential for increasing polymeric-based artificial joints' operational durability and performance, ensuring better reliability and functionality in biomedical applications.

Surface texturing has received the attention of tribologists across numerous industry sectors for the past two decades, provoking a breadth of experimental and theoretical publications aimed at deriving optimum texture parameters within the context of a given application. This technique modifies surface texture to reduce friction, control wear, and improve load capacity [17, 18]. In other words, surface texturing has been shown to reduce the friction coefficient by enhancing hydrodynamic lubrication between contacting surfaces. The role of surface texturing in component design is ever-increasing; the ability to influence the tribological behaviour between two frictional surfaces is the driving factor for introducing deterministic surface features for creating functional surfaces. extures, such as micro-dimples or grooves, can trap lubricants and create pressure gradients that improve lubrication and reduce direct surface-to-surface contact, thereby mitigating wear. This technique not only reduces wear particle generation but also minimizes the risk of prosthetic loosening, a critical issue in both orthopedic and spinal implants. However, challenges remain in understanding the mechanistic functional details of texturing in tribological situations and developing well-defined processes for producing optimised deterministic textures [19]. The benefits of textures can be derived from a hydro-dynamic effect created by a diverging-converging wedge of the lubricant-filled texture cavity, a secondary supply of lubrication, and the entrapment of wear debris [20, 21].

Many analytical studies have evaluated the effects of texture geometry and density on the resultant pressure generation and frictional forces. For instance, Teo et al. [22] demonstrated friction reduction of up to 25% using textured surfaces compared to untextured ones. Wei et al. [23] used computational fluid dynamics to analyse the impact of dimple geometry on pressure generation. In another study, Gherca et al. [24] found that texture geometry, particularly cell number, depth, and density, significantly influences load support and friction on parallel bearings. Ronen et al. [25] investigated the application of surface textures for a piston/cylinder reciprocating contact. An optimum aspect ratio (depth/diameter) was between 0.1 and 0.18. The texture density was shown to have a minimal effect, with only a 7% change in friction force between densities of 5% and 20%.

Furthermore, it was found that friction reduced sharply with increased pores until a saturation point was reached, after which the reduction in friction force became negligible [25]. Ramesh et al. [26] employed computational fluid dynamics (CFD) to analyse the effect of circular textures. Although the friction force increased concerning load, it was shown that it produced no shift in the optimal texture parameters. Zhou et al. [27] found that texture density was influenced by sliding speed. They stated that a higher density texture coverage of between 16 and 22% at lower speeds was optimal. However, as sliding speed increased, the optimal texture density reduced to that of 10–12%; the effect of surface texturing on the tribological performance of thurst bearings was investigated by Wang et al. [28]. They observed a significant difference between experimental and numerical results. They attributed this to external influences not considered within the numerical model, such as contact pressure and the generation of tribal-chemistry products that led to a film formation that further reduced the friction.

Assessing the application of surface textures for improving tribological performance is primarily investigated through experimental studies and have demonstrated significant friction reduction in cutting tools up to 34.5%, piston-ring and cylinder liners up to 82%, seals up to 65%, and bearings up to 18% [17]. Laser surface texturing is widely used to create micro-patterns, particularly dimples and grooves on surfaces [29]. Kovalchenko et al. [30] evaluated the effect of Laser Surface Textures (LST) on transitions in lubrication regimes, where six specimens were tested in a pin-on-disc experiment. The resultant Stribeck Curve for ground, polished, and best-textured specimens demonstrated the influence of surface textures by shifting the transition of lubrication regimes to higher loads and lower speeds for a low-viscosity lubricant. This phenomenon was also observed by Borghi et al. [31] in a pin-on-disc experiment on nitride steel. Replicating starved lubrication conditions showed that substantial reductions in the coefficient of friction (COF) were achieved by introducing surface textures.

Furthermore, a Stribeck analysis revealed transitions between lubrication regimes within the Stribeck curve. Hu et al. investigated the effect of surface textures employed within the boundary lubrication regime on the tribology performance. [32]. The experimental investigation measured the impact of texture density on the tribological performance under lubricated conditions of high and low-viscosity lubricants. They found that the dimple density profoundly affected various operational parameters such as load and lubricant type. Sliding tests employing a lubricant of low viscosity showed that all textured samples out-performed un-textured surfaces under low load conditions, with a density of 8.5% observed to be optimum. Increasing the load under identical lubrication conditions showed an increase in the optimum density, rising to 35% [32].

Although there are many reported studies in the literature, only some have discussed the impression of hydrodynamic pressure, and its mechanism still needs to be fully understood. Due to oversimplifications and unaccounted variables, existing analytical models often need to accurately predict the frictional performance of textured surfaces under hydrodynamic conditions. Therefore, this paper presents the results of the frictional performance of a range of dimple-shaped surface textures with a theoretical model developed to confirm the presence of hydrodynamic pressure in experimental conditions. The novelty of this work lies in two key aspects. Firstly, an innovative theoretical model has been developed that more accurately captures the presence of hydrodynamic pressure under the specified experimental conditions. Unlike previous models, this approach incorporates detailed texture parameters such as the diameter and depth of the strokes. Secondly, a robust multiple regression model has been constructed based on empirical data, enabling the prediction of frictional performance across a wide range of texture parameters. Tests were carried out, and empirical results were employed to develop a multiple regression model to predict the resultant friction over a wide range of texture parameters. A model was developed to predict the friction for textured surfaces. The two models were compared by standardising the data and assessing the effect of each texture parameter. Finally, the form of the empirical friction trace was compared to that of the theoretical model, which showed a strong correlation. The obtained insight in this study is particularly valuable in the design of novel materials for applications where stable lubrication is critical, such as in biomedical implants or high-precision industrial components.

## Materials and methods

### Materials

**Fig. 1:**
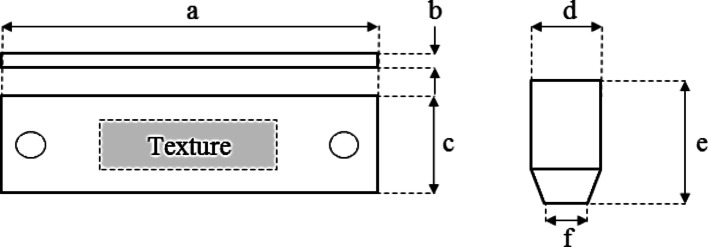
Schematic of the Lower Specimen (flat plate, PEEK) and upper specimen (cylindrical pin, PEEK). **a** 60 mm, **b** 4 mm, **c** 25 mm, **d** 8 mm diameter, **e** 20 mm, **f** 5 mm

Poly-ether-ether-ketone (PEEK) 450G (Invibio Ltd.) was utilised for both upper and lower specimens (shown in Fig. 1). The lower sample was a rectangular section of 60 × 25 mm^2^ and 4 mm thick. The upper specimen was an 8 mm extruded rod with the contact end turned down to a 5 mm diameter. The detailed dimensions are presented in Fig. 1. The physical properties of PEEK (450G) include a modulus of elasticity of 3.6 GPa and a hardness of 20 HV, making it suitable for both load-bearing and tribological testing.

The surfaces were polished to an average surface roughness (R_a_) of 0.12 µm ± 0.002 µm using 600, 800, 1000, and finally 1200 grit silicone-carbide (SiC) paper. Surface roughness measurements were measured on an Alicona G5 InfiniteFocus. The lower surfaces were polished by fastening the plate to a ground steel block. The polishing paper was attached to a flat ground surface and then polished by sliding the steel block over the polishing paper. Both sides of the plate were polished, ensuring the surfaces were parallel. The upper specimens were polished using a milling machine. Upper samples were inserted into a purpose-made holder and located in the chuck of the milling machine. A SiC polishing block was held in the vice, and a dial test indicator was used to ensure the block was true to the spindle.

The specimens were prepared by washing them with water and soaking them in an ultrasonic bath maintained at 40 °C for 5 min. They were then dried with a lint-free cloth and soaked in acetone for a further 2 min; once removed, they were left to air dry.

### Surface texturing

**Fig. 2:**
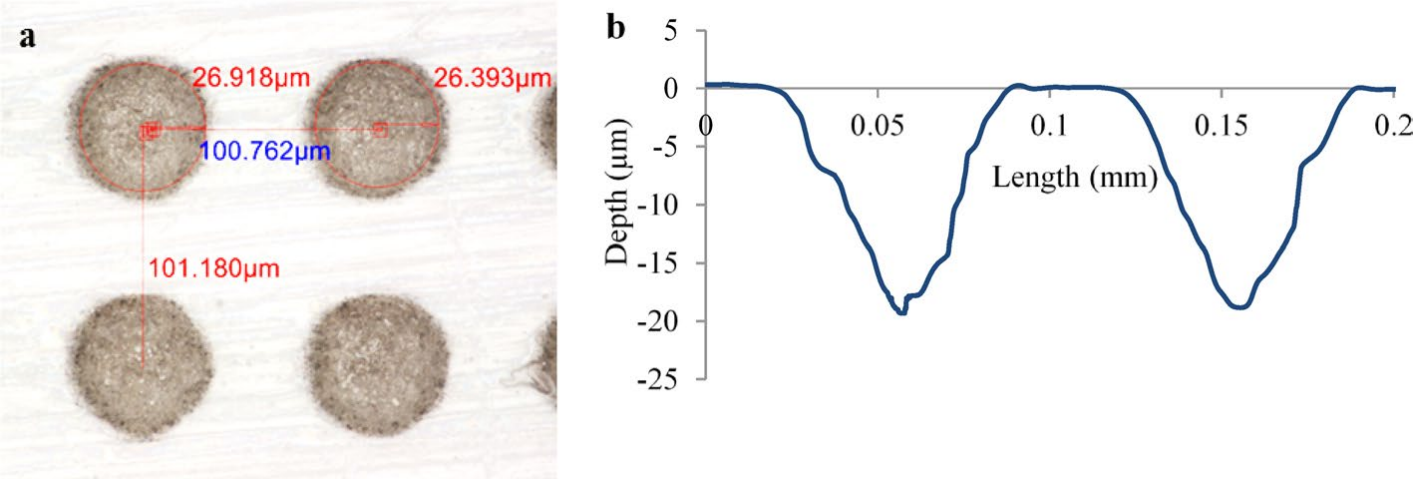
**a** Representative image of 50 µm diameter texture dispersed over the surface at a density of 20%. **b** Profile image of 50 µm texture with a depth of 20 µm

Cylindrical cavities were created using a Yb-doped femtosecond laser (Amplitude Systems). The textures were applied to the lower specimen (plate) in column arrays aligned to the sliding direction. Cavity diameters of 50 µm, 100 µm, and 200 µm were dispersed over the surface at a density of 20%, with each diameter being studied at three different depths of 5 µm, 10 µm and 20 µm. The representative image in Fig. 2a and b exhibit a 50 µm diameter cavity dispersed at a density of 20% and the corresponding profile for 20 µm depth, respectively. The density of 20% was applied following previous studies relating to applying surface textures to low-moduli materials. Tests were conducted for 1800s with friction force measurements taken at one-second intervals. Nine individual diameter-depth combinations were evaluated, with each test repeated three times, equating to 27 tests plus three tests for a control surface (un-textured).

### Experimental parameters

**Fig. 3:**
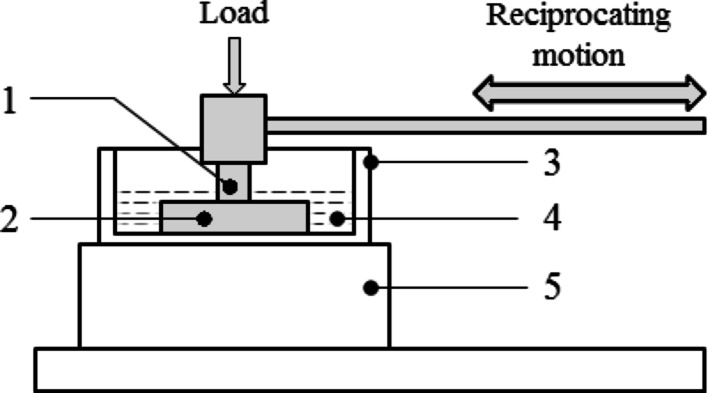
Experimental layout of TE77 tribometer. (1) upper specimen (un-textured) (2) lower specimen (textured/un-textured), (3) lubricant bath, (4) lubricant (reagent grade purified water), (5) heater block

Short-duration reciprocating sliding tests were carried out using a TE77 (Pheonix Tribology Ltd, Kent UK) High-Frequency Reciprocating Test Rig. A schematic of the experimental arrangement is shown in Fig. 3. Empirical examinations were performed under a pin-on-plate conformal contact. The lower sample was fixed to the base of the lubricant bath; the reciprocating motion was provided by a scotch yoke mechanism set at a frequency of 1 Hz across a stroke length of 7.4 mm and was subjected to an applied load of 75 N (representative of the physiological forces experienced in load-bearing joints like the hip or knee during moderate activity), corresponding to a contact pressure of 3.5 MPa and siding speed of 1500 rpm (simulate the high-frequency cyclic movements experienced in articulating joints). Tests were performed under fully flooded lubrication conditions. The lubricant was reagent-grade purified water, initially heated to 37 °C (normal human body temperature) and maintained at this temperature throughout the test. The test conditions were carefully selected to simulate an environment relevant to biomedical applications, particularly focusing on conditions similar to joint replacements or other implantable devices subjected to cyclic loading in the human body.

## Experimental results

**Fig. 4:**
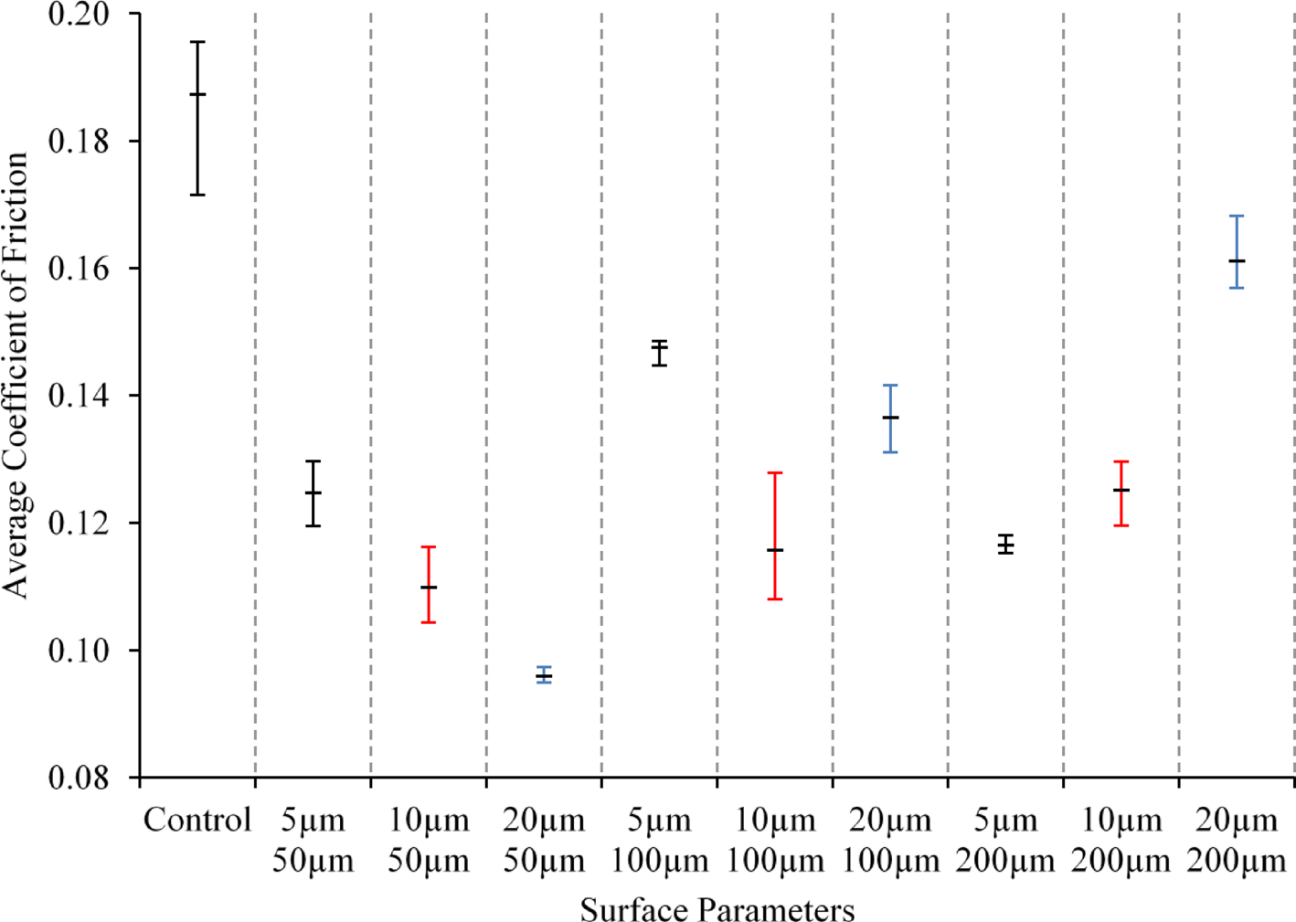
The variation of average COF with changing surface parameters—diameter and depth—for a PEEK frictional Couple. Sliding at a reciprocating frequency of 1 Hz under a load of 75N in water-lubricated conditions maintained at a temperature of 37 °C

Figure 4 shows the variation in the average coefficient of friction corresponding to the surface structure. The average coefficient of friction was calculated as the mean value taken across the entire test duration. The upper and lower error bars denote the recorded maximum and minimum friction force, and the mid-point represents the average friction force across the three test repetitions.

The coefficient of friction for all textured surfaces outperformed the untextured control specimen, which attained an average coefficient of friction of 0.187. It is shown that the COF decreased in all textured specimens. The lowest average coefficient of friction observed was that of a textured surface with a diameter of 50 µm and a depth of 20 µm, recording an average friction force of 0.096. The highest friction force registered for a textured surface was from a diameter and depth combination of 200 µm and 20 µm, respectively. Furthermore, it can be observed that the COF increases by increasing the texture diameter, which can be related to the average friction force generally increasing with an increase in texture diameter. Zhang et al. [33] reported that texture depth and diameter are crucial in determining COF and wear reduction. Although such trends and the impact of the surface texture on COF have been reported in the literature [34, 35], similar trends cannot be determined regarding texture depth, where the response according to the depth parameter fluctuates with variations in texture diameter.

## Experimental model

A regression model based on the experimental data was developed to investigate the influence of the depth and diameter parameters on the resultant friction force. Implementing an experimental model was motivated by the need to evaluate the parameter effects through the friction force over a broader data set and for adequate comparison with an analytical model.

**Fig. 5:**
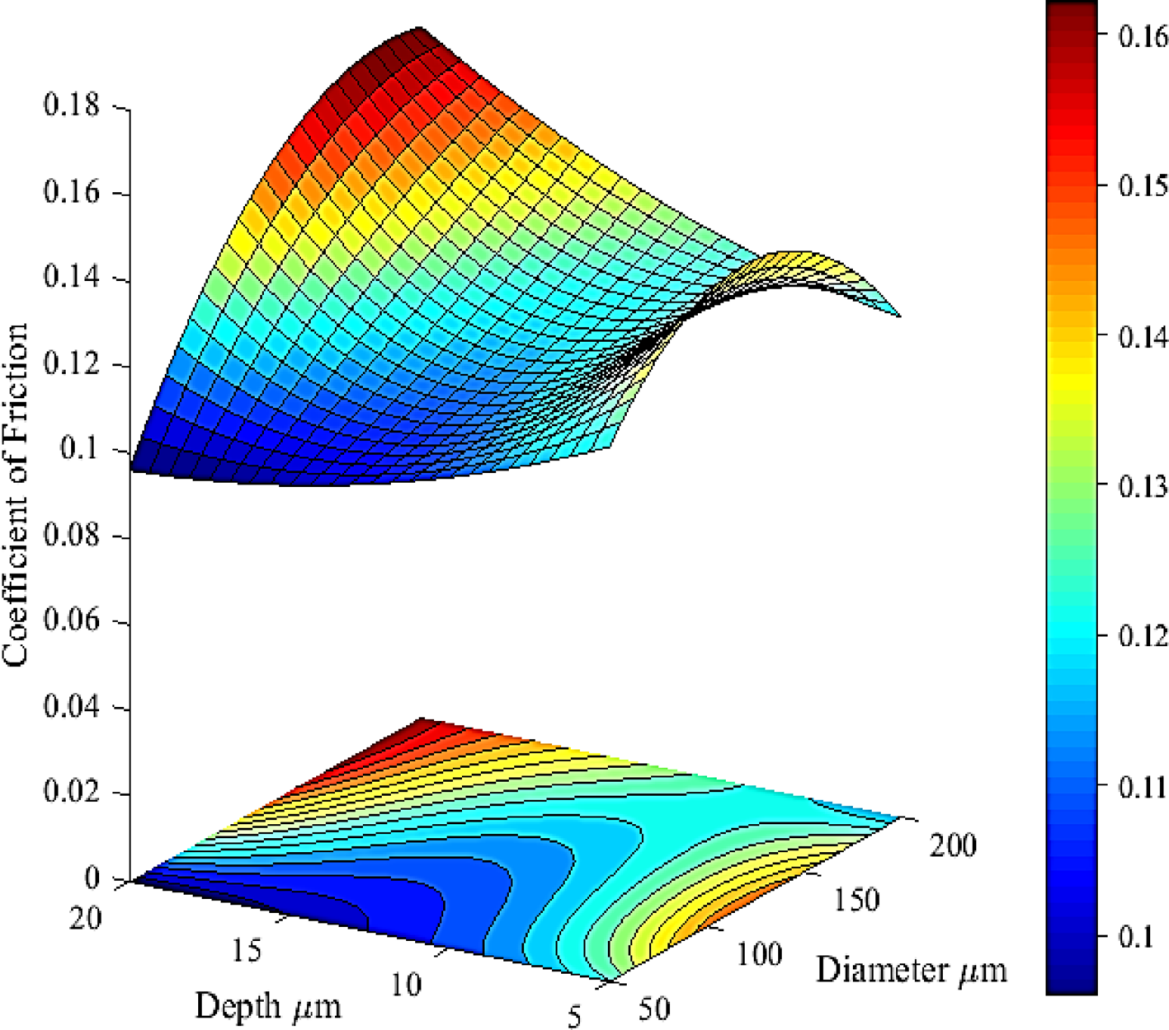
3D plot of the regression model, showing the effect of diameter and depth combinations has upon the average coefficient of friction

The model was derived by fitting a curve to the experimental data across the diameters at various depths. A similar procedure was applied to obtain the curve fitting across different depths at multiple diameters. In both cases, a 2nd-order polynomial trend line fit the data well. The equations representing these curves were derived from the trend-line functions, and the complete polynomial coefficients were obtained using the LINSET function. The model, shown in Fig. 5, predicts the average coefficient of friction for texture diameters ranging from 50 to 200 µm in 25 µm increments and for depths from 5 to 20 µm, increasing in 2.5 µm increments.

## Analytical modelling

**Fig. 6:**
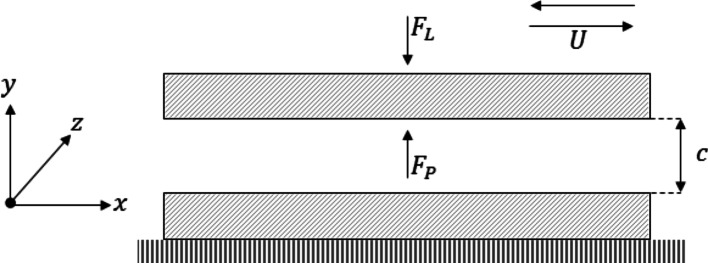
Model schematic and coordinate scheme of two conformal surfaces separated by a thin film of lubricant, c. The upper surface travels at a reciprocating velocity, U, and the lower surface is fixed. An applied load, F_L_ is opposed by a force deriving from hydrodynamic pressure, F_P_

A two-dimensional model was developed to evaluate the presence of hydrodynamic phenomena and predict the friction force with the experimental parameters. The schematic in Fig. 6 illustrates the model layout, whereby a thin fluid film thickness separates two conforming surfaces, $c$. A force is applied to the top surface, which is counteracted by an equal force generated by the hydrodynamic pressure of the fluid. The bottom textured surface is fixed, and the upper surface travels in a reciprocating motion at a velocity, $U$. Appropriate boundary conditions are essential for accurate dynamic simulation, especially in complex geometries, as they ensure compatibility with external constraints and influence the results' quality. Therefore, considering the geometry presented in Fig. 6, some boundary conditions must be considered, such as pressure of the hydrodynamic forces, motion effects, texture geometry, film thickness, force balances, and friction force. The governing equations for each part and considered assumptions are clarified subsequently.

The Reynolds equation, Eq. 1, was assumed to model the generated hydrodynamic pressure over three following texture pores within a single stroke length.

1 $$\left\{{h}^{3}\frac{{\partial }^{2}p}{\partial {x}^{2}}\right\}+\left\{{h}^{3}\frac{{\partial }^{2}p}{\partial {z}^{2}}\right\}=6\mu U\frac{\partial h}{\partial x}+12\mu \frac{\partial h}{\partial t}$$

where p is the pressure within the fluid film, h indicates the film thickness, U implies the velocity of the moving surface and η represents the dynamic viscosity of the fluid. To solve the Reynolds equation, dimensionless expressions (Eq. 2) were introduced and substituted into Eq. 1, which resulted in Eq. 3 as below:

2 $$X=\frac{x}{{r}_{p}};Z=\frac{z}{{r}_{p}}; H=\frac{h}{hp}; P=\frac{p}{{P}_{a}};T=\frac{t}{{T}_{o}};U=\frac{u}{{r}_{c}\omega };$$

where, X and Z are dimensionless lengths in the x and z directions, respectively, and H, P, T, and U indicate dimensionless film thickness, pressure, time, and velocity, respectively. r_c_ us characteristic radius and ω in angular velocity.

3 $$\left({H}^{3}\frac{{\partial }^{2}P}{\partial {X}^{2}}\right)+\left({H}^{3}\frac{{\partial }^{2}P}{\partial {Z}^{2}}\right)=\psi U\frac{\partial H}{\partial X}+\xi \frac{\partial H}{\partial T}$$

where,

4 $$\psi =\frac{6\mu {r}_{c}\omega {{r}_{p}}^{3}}{{h}_{p}{P}_{a}}; \xi =\frac{12\mu }{{T}_{o}{P}_{a}}$$

**Fig. 7:**
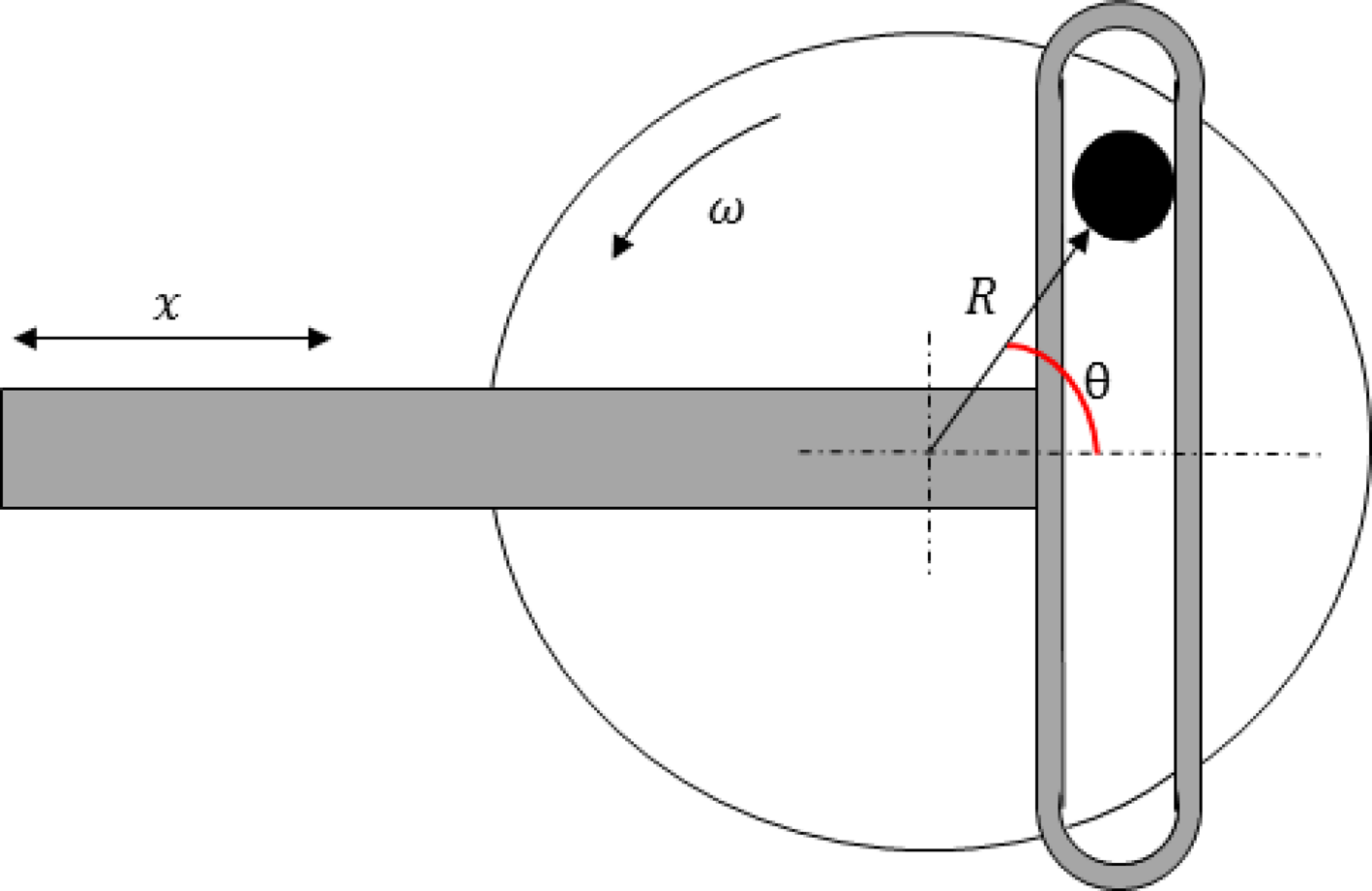
The schematic of the Scotch yoke mechanism

The motion of the upper sample was driven by a scotch yoke mechanism providing simple harmonic motion for converting rotary motion to linear motion or vice versa [36]. The equation of motion for the mechanism is modified for the analytical model. As illustrated in Fig. 7, the conversion of constant rotation to reciprocating motion with a displacement of x, relative to the angle, θ, and radius, R, the relationship to computing the velocity, U, is provided in Eq. 5.

5 $$U=\frac{dx}{dt}=\omega Rcos(\omega t)$$

**Fig. 8:**
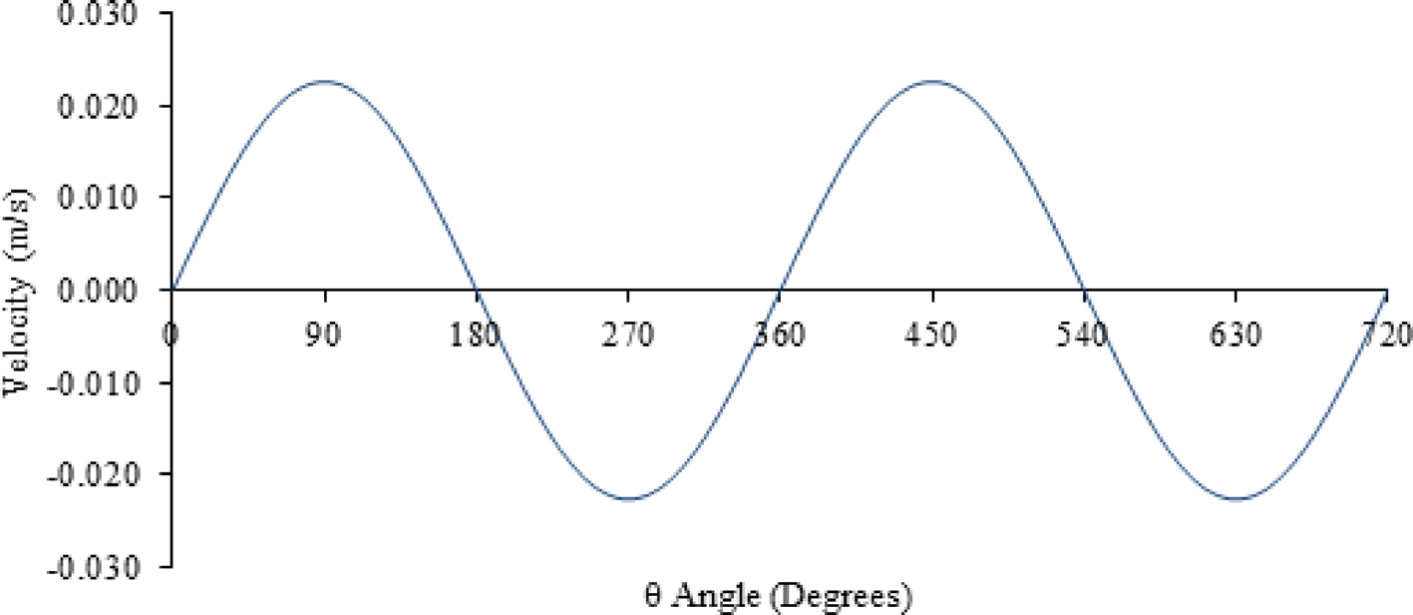
Velocity profile produced by a scotch yoke mechanism with a radius R equivalent to the experimental stroke length, rotating at 60 RPM over a time scale of 2 s. The example illustrates 2 complete rotations (720°) corresponding to 4 strokes contained within 2 reciprocating cycles

Applying the experimental parameters to the analytical study, where R is half the stroke length, ω is taken as 60 RPM, to provide a 1 Hz reciprocating frequency and t is the time to be analysed. Figure 8 represents the resultant velocity as a function of θ.

**Fig. 9:**
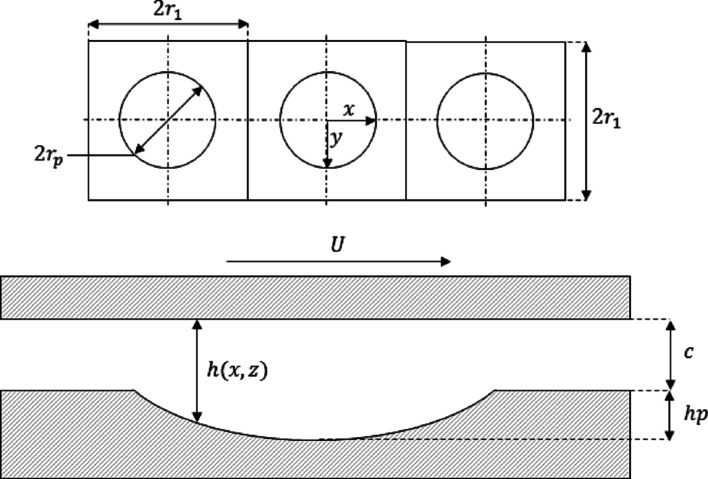
Surface geometry and notation. Plan view of 3 consecutive textures indicating texture cell length (2r_1_), texture diameter (2r_p_), and coordinate scheme. The bottom figure shows a cross-section across one textured cell separated by an initial gap, c. Where h (x,z) is the instantaneous film thickness and hp is the depth of the textured pore

Figure 9 shows the surface geometry, where a plan view of three consecutive textures indicates texture cell length (2r_1_), texture diameter (2r_p_), and coordinate scheme. A cross-section of one textured cell is also shown; the geometry is separated by an initial gap, c, where hp is the depth of the textured cavity and h($x,z$) is the instantaneous film thickness. To simplify the model and reduce computation time, it was assumed that pressure generated along the x-axis was sporadic with stroke length. The pressure was thought to repeat itself across adjacent textures in the z-direction. For these reasons, only one stroke across three consecutive textures with a width of one singular pore column was analysed.

The local film thickness varied across the stroke length where the boundary conditions are expressed through Eq. 6 as below:

6 $$h=\left\{\begin{array}{c}ct+{h}_{px} \left(x,z\right) when {x}^{2}+{z}^{2} < {r}_{P}\\ ct {x}^{2}+{z}^{2} \ge {r}_{P}\end{array}\right\}$$

where $ct$ is the film thickness across the stroke owing to a low-frequency sinusoidal squeeze motion and $h{p}_{xz}$ is the instantaneous texture depth relative to $x$ and $z$. The textures were modelled as a spherical cap with a depth of *h*_*p*_, and a length of *2r*_*p*._ The boundary conditions were applied to and instantaneous film thickness in dimensionless form was expressed as Eq. 7:

7 $$ H(X,Z) = \left[ {\begin{array}{*{20}l} {\frac{{ct + \sqrt {\left( {h_{p}^{2} + r_{p}^{2} } \right)^{2} - (x^{2} + z^{2} ) - \frac{{r_{p}^{2} - h_{p}^{2} }}{{2h_{p} }}} }}{{h_{p} }}} \\ {Ct} \\ \end{array} } \right]\begin{array}{*{20}c} {X^{2} + Z^{2} < 1} \\ {X^{2} + Z^{2} \ge 1} \\ \end{array} $$

The relevant boundary conditions were applied concerning the assumption of a cavitation region at the point where fluid entered the textured pores. The Reynolds (also known as the Swift-Steiber) boundary condition implies that the pressure reduces to zero at the boundary of the cavitation zone, the Reynolds boundary condition was applied for each iteration [25].

For the system to be in equilibrium the force generated from hydrodynamic pressure (F_P_) must be opposite and equal to the normal force (F_L_), as illustrated in Fig. 6 and expressed in Eq. 8.

8 $${F}_{L}-{F}_{P}=0$$

However, the applied force generates various contact pressures, owing to the dimensions of the textured cells being a function of the diameter. To account for this, a constant random applied force was scaled to reflect the variations in texture dimensions, resulting in each textured array being subjected to an identical contact pressure.

The dimensionless vertical force deriving from hydrodynamic pressure was computed by integrating the pressure across the area according to Eq. 9;

9 $$\overline{{F }_{p}}=\iint pdxdz/{p}_{a}$$

And according to Eq. 10, the normal force was expressed in dimensionless form as;

10 $${F}_{L}=Applied \,Load/{p}_{a}$$

Finally, the friction force due to the shearing of fluid between the opposing surfaces was calculated by integrating the resultant shear stress (τ) over the area, according to Eq. 11 as below.

11 $$F=\underset{0}{\overset{L}{\int }}\underset{0}{\overset{B}{\int }}\tau dxdz$$

where, shear stress can be calculated through Eq. 12 as below:

12 $$\tau =\left(\frac{\mu U}{h}\right)+\frac{\partial p}{\partial x}\frac{h}{2}$$

With substituting Eq. 12 in 11, Eq. 13 can be written where, it is presented in a dimensionless form the friction force was expressed as;

13 $$Ff=\underset{0}{\overset{L}{\int }}\underset{0}{\overset{B}{\int }}\left[\left(\frac{\mu U}{h}\right)+\frac{\partial p}{\partial x}\frac{h}{2}\right]/{P}_{a}$$

**Fig. 10:**
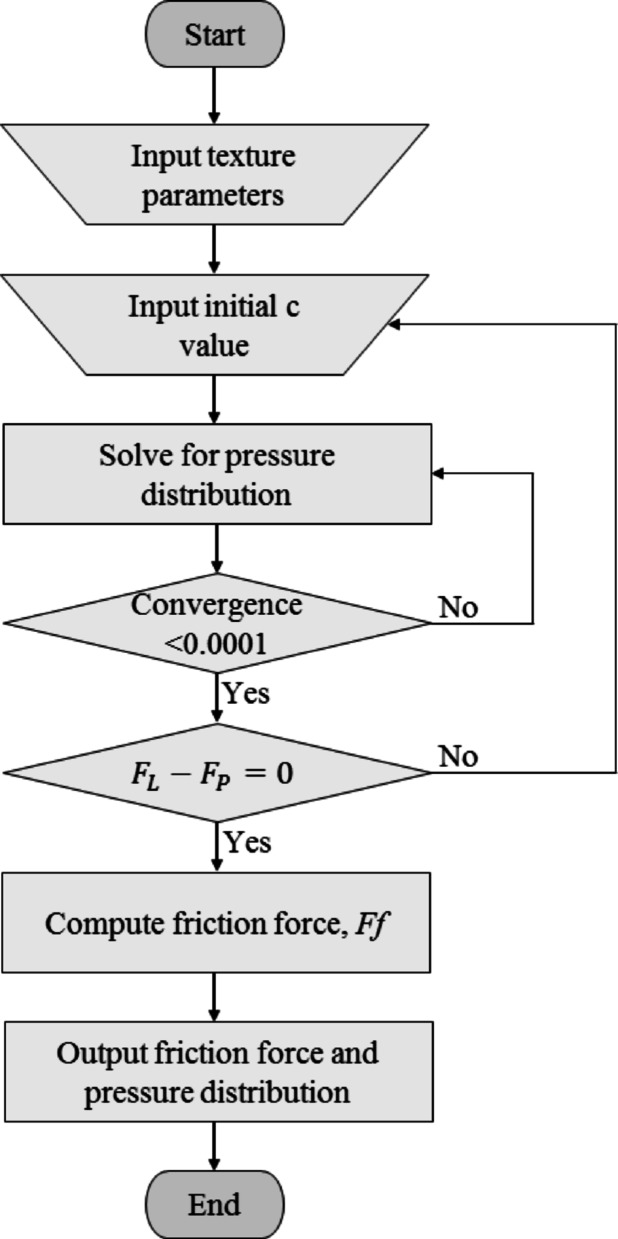
Flowchart identifying the computation procedure to determine the force between applied load and hydrodynamic pressure through to the calculation of friction with the specified surface texture parameters

The Reynolds equation was solved by utilizing the finite difference method. The surface was discretized into a fine, uniform grid structure. The Jacobi method was the iterative procedure used to achieve convergence, shown in Eq. 14. The calculation was stopped once the convergence criteria had been met, which should be lower than 0.0001 as in Eq. 14, where the complete computation procedure is illustrated in Fig. 10, where the friction force and pressure distribution were calculated.

14 $$Convergence \,Criteria=\frac{P\left(k\right)-P(k-1)}{P(k-1)}=<0.0001$$

## Results and discussion

### Hydrodynamic pressure

**Fig. 11:**
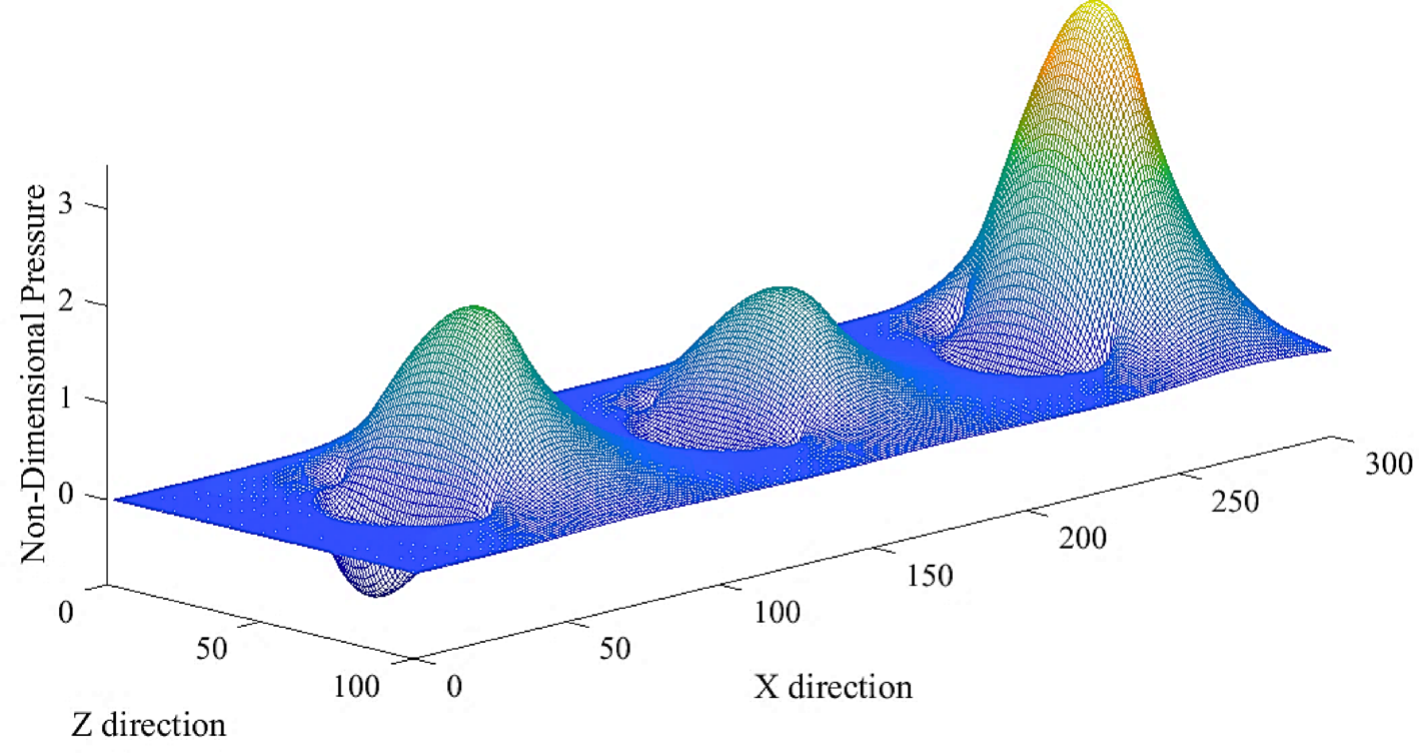
dimensionless pressure distribution within one reciprocating stroke across three consecutive textures with a diameter of 50 µm and depth of 10 µm

The developed theoretical model predicted the produced hydro-dynamic pressure and friction force with the parameters set. Figure 11 shows the 3D variation of hydro-dynamic pressure across the stroke in the x and z directions. It can be observed that the pressure differs throughout the stroke, which can be related to the variation in relative motion, velocity, the squeeze film effect, and subsequent changes in film thickness. In other words, as the relative motion and velocity changes, it affects the hydrodynamic pressure distribution within the fluid film, which can be interpreted from Fig. 11. Additionally, the squeeze film effect, which occurs when the fluid is compressed or expanded, leads to fluctuations in pressure. These combined factors cause the film thickness to vary, further influencing the pressure dynamics throughout the stroke [37, 38].

**Fig. 12:**
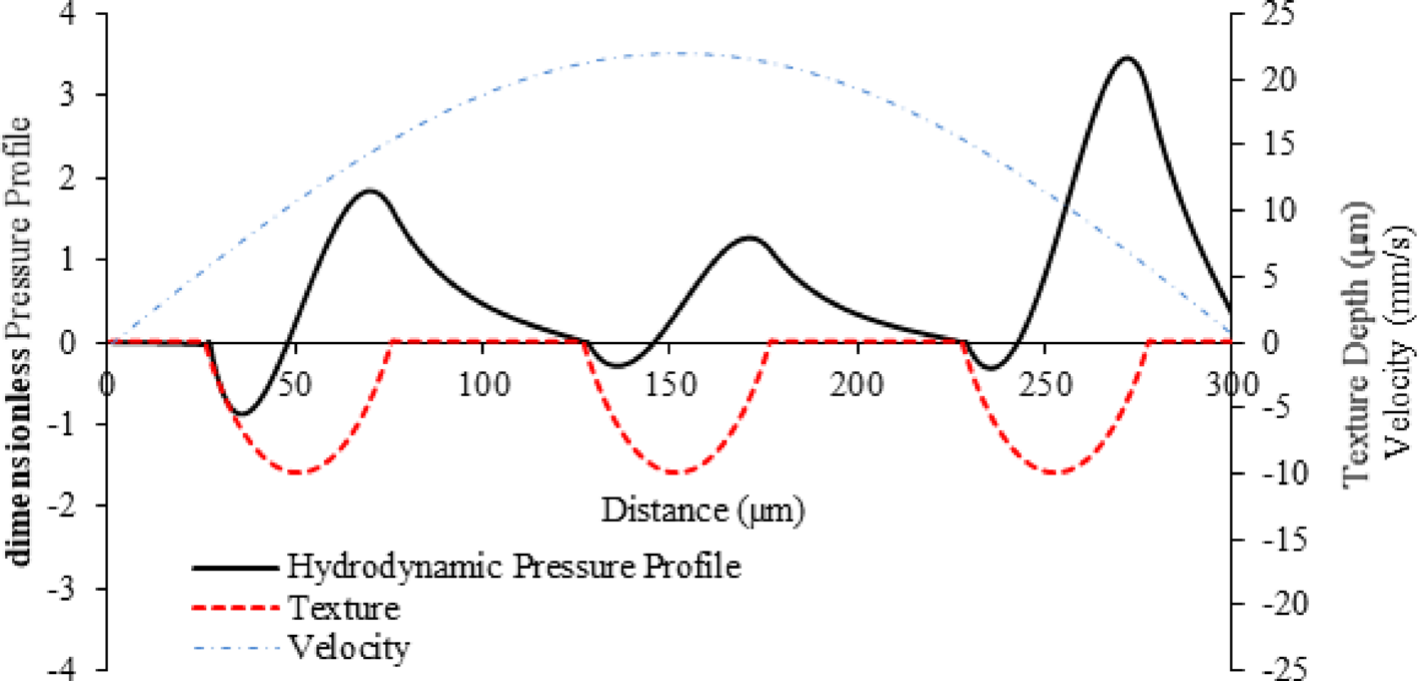
Hydrodynamic pressure distribution plotted over 1 reciprocating stroke moving at velocity (v) across 3 consecutive textured pores with dimensions of 50 µm diameter and a depth of 10 µm

For a detailed study, the 2D variation of the hydrodynamic pressure along the distance (x-direction) and motion velocity is plotted and presented in Fig. 12. It is shown that as the velocity increased the pressure produced was reduced, which is attributed to an increase in film thickness. This occurs because higher velocities enhance the hydrodynamic lift, which causes the fluid film to thicken. A thicker film reduces the contact and friction between surfaces, decreasing the pressure generated within the fluid film. Thus, the increased velocity results in a higher film thickness and consequently lower pressure. However, as the velocity reduced the pressure developed towards the end of the stroke increases. This phenomenon is influenced by the decrease in velocity at the extremity of the stroke which minimises the gap between the two surfaces. As the surfaces come closer together, the fluid film is compressed, leading to an increase in hydrodynamic pressure. The decreased velocity allows less time for the fluid to escape from the gap, resulting in a higher-pressure accumulation. Thus, the combination of reduced velocity and a narrower gap between the surfaces elevates the pressure at the end of the stroke. Subsequently, the bulk fluid at this point pressurises owing to a squeeze effect.

**Fig. 13:**
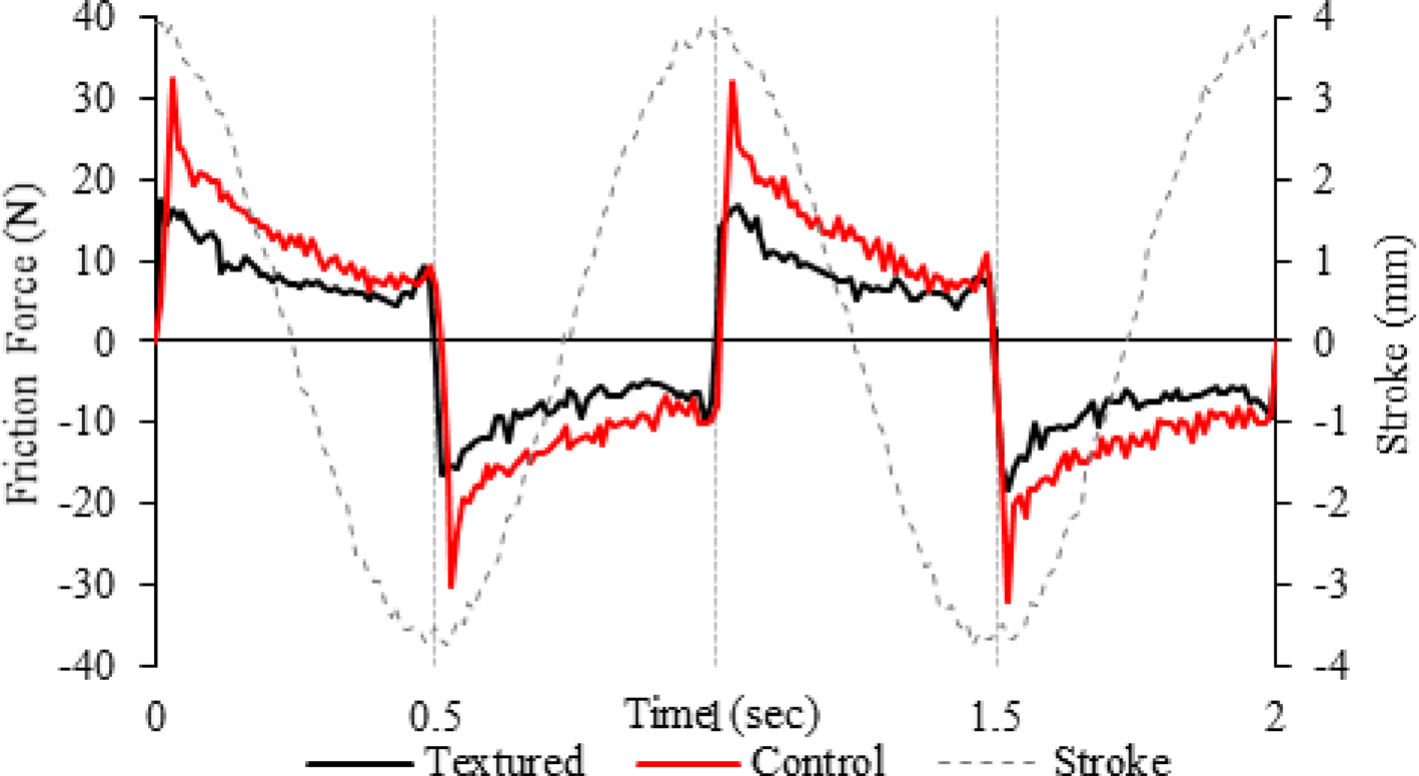
Friction force and Stroke length vs. Sliding time for a control (un-textured) and a textured surface. Friction data captured at 100 Hz over 2 s. Tests were conducted at 1 Hz reciprocating frequency, a load of 50N with water lubrication maintained at 37 °C

For validation of this phenomenon, the friction force as a function of time for 4 reciprocating strokes for a control (un-textured) and textured surface of 100 µm diameter and 10 µm depth is exhibited in Fig. 13. The high-speed data was captured at a frequency of 100 Hz over 4 s, and 900 s into the experimental tests conducted. The drop in friction force can be detected at the end of the stroke and the subsequent presence of hydrodynamic and squeeze film effects. Here, the asymmetry in the friction force is a clear sign of lubrication phenomena, enhancing the tribological performance of both control and textured surfaces towards the end of the stroke. Therefore, Fig. 13 suggests the notion of a hydrodynamic and squeeze film effect by the general form of the friction trace. If these effects were not present the friction trace would be approximately symmetrical around the centre of the stroke because the friction force would be primarily governed by contact mechanics, where the friction force depends linearly on the normal load and the coefficient of friction; however, both the control and textured surfaces exhibit a drop-in friction force towards the end of the stroke. The textured surface might enhance the hydrodynamic and squeeze film effects by trapping more lubricant and creating micro-reservoirs, maintaining a more effective lubrication regime throughout the stroke. This would lead to a more significant drop in friction force towards the stroke's end than a smooth surface.

**Fig. 14:**
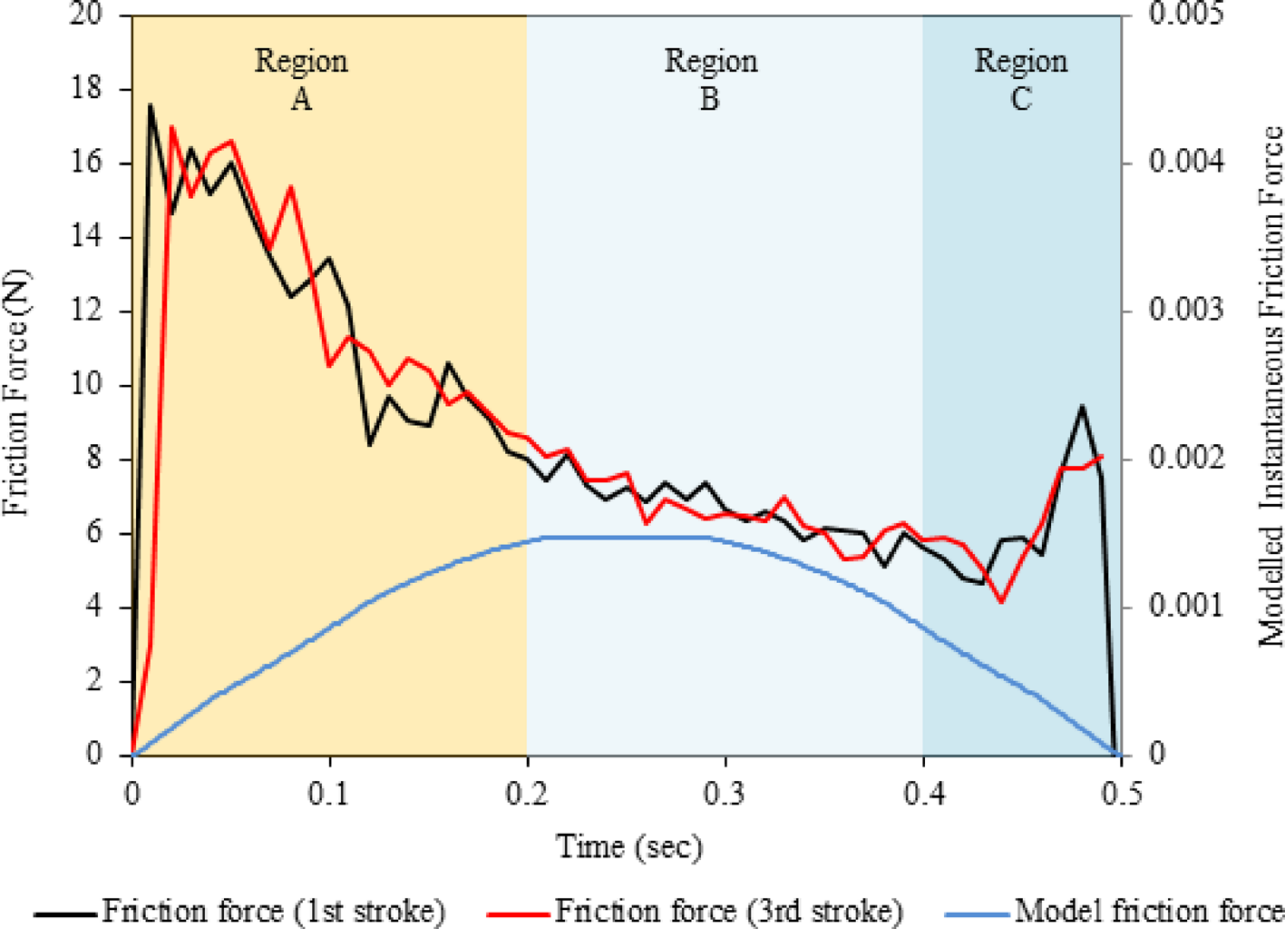
Experimental friction force for a textured surface vs the analytically modelled instantaneous friction force for a textured surface of 100 µm diameter and 10 µm depth. The plot highlights the three regions of tribological activity expected within a reciprocating contact where Region A: Boundary/mixed lubrication, Region B: Hydrodynamic and squeeze film lubrication, and Region C: Mixed/boundary lubrication

Figure 14 compares the instantaneous friction force across one stroke for the analytical model and the captured high-speed friction force data for the first and third stroke of the textured surface shown in Fig. 13. Using Eq. 13, the instantaneous friction force for the analytical model was calculated, across the stroke length and agreed with the velocity profile. Three regions that describe the tribological processes within a single stroke are annotated on the plot.

- Region A: The friction initially rises due to the limiting friction force that the force in the direction of motion is required to overcome to facilitate the transition from static to kinetic motion. Once sliding commences, the velocity is insufficient to generate a stable fluid film. This gives rise to surface interaction as local asperities contact, creating a boundary/mixed lubrication regime.
- Region B: The hydrodynamic and squeeze effect stage as the friction force begins to fall owing to the formation of a fluid film.
- Region C: As the fluid film thickness decreases, velocity reduces giving rise to asperity contact between the opposing surfaces. Furthermore, wear debris not expelled from the contact zone is entrained and trapped between the surfaces. At this point, the lubrication regime reverts to a mixed/boundary state.

Divergence can be detected within all regions by comparing the experimental and analytical friction forces. This is attributed to the tribological activity previously described, which needs to be accounted for within the model, such as surface roughness contact mechanics and boundary lubrication. However, region B shows a better fit in their respective form as both the experimental and theoretical friction forces decrease. This illustrates that within the hydrodynamic regime of a reciprocating stroke, the system supports the notion of hydrodynamic pressure and squeeze film action.

### Effect analysis

To fully understand the individual effects of texture depth and diameter, the following analysis incorporates empirically derived, statistically modelled values of the coefficient of friction. The friction force is obtained by multiplying the coefficient of friction by the experimental load of 75 N. The average is taken across each diameter at various depths on computing the friction force. The same method was employed to calculate the depth effect, whereby the average was taken across each depth over multiple diameters.

To compare the modelled dimensionless and experimentally derived friction force, the results were standardised according to Eq. 15.

15 $$\beta =\frac{Variable-\overline{x}}{\sigma }$$

where, the friction force is the variable, $\overline{x }$ and σ are the population mean and standard deviation, respectively, and β is the standardised form of friction force.

**Fig. 15:**
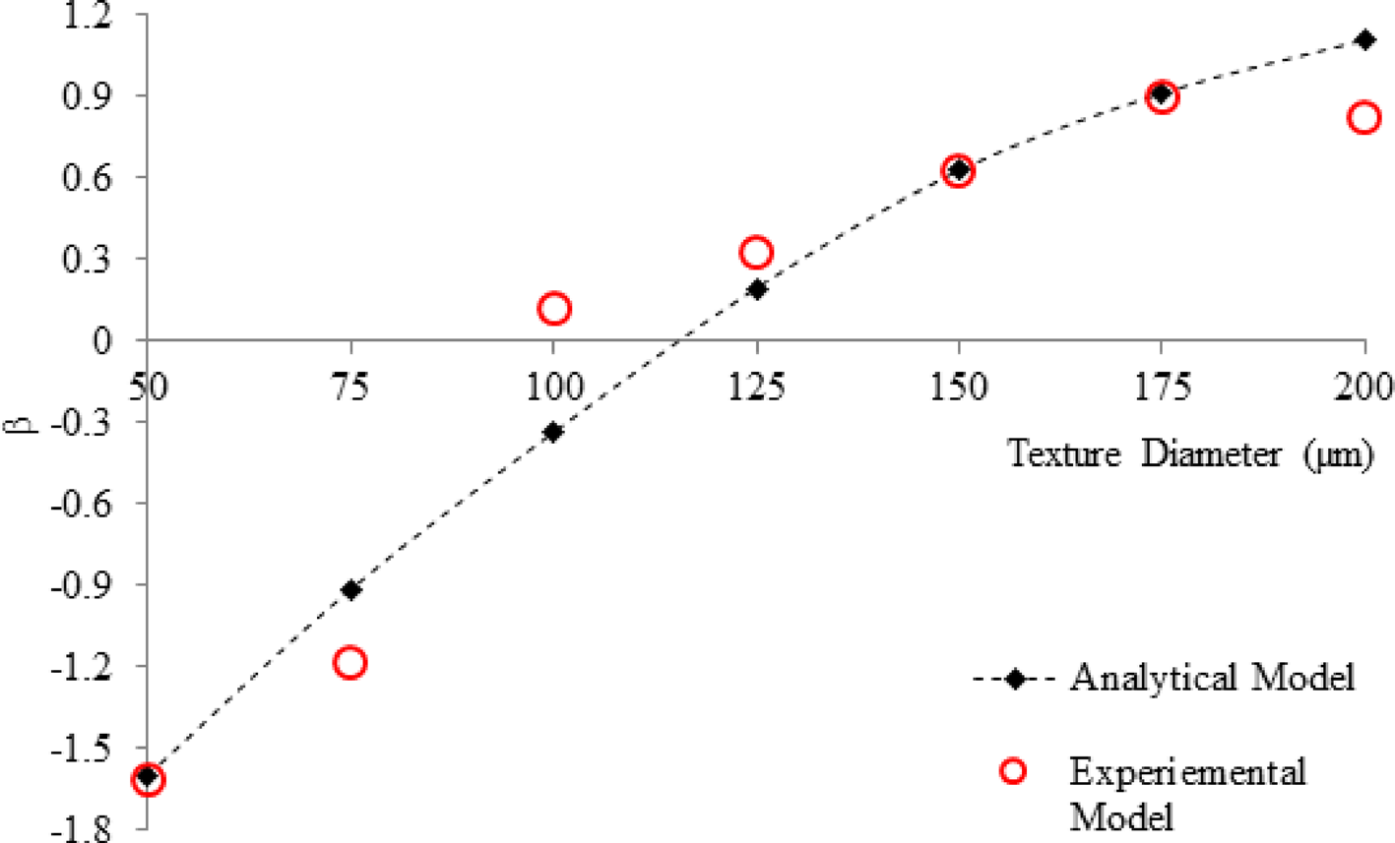
Comparing the standardized friction force for the theoretical and experimental model at various depths in the range from 50 to 200 µm at 25 µm intervals

The comparison of the analytical model and experimental results of the effect of texture diameter is presented in Fig. 15. As can be seen, a good agreement was achieved between the theoretical and experimental models, where increasing the diameter increased friction force. Increasing the diameter generally leads to an increase in friction force due to a larger contact area, changes in pressure distribution, and more significant surface interactions, which confirms that they were well-considered in the theoretical model.

**Fig. 16:**
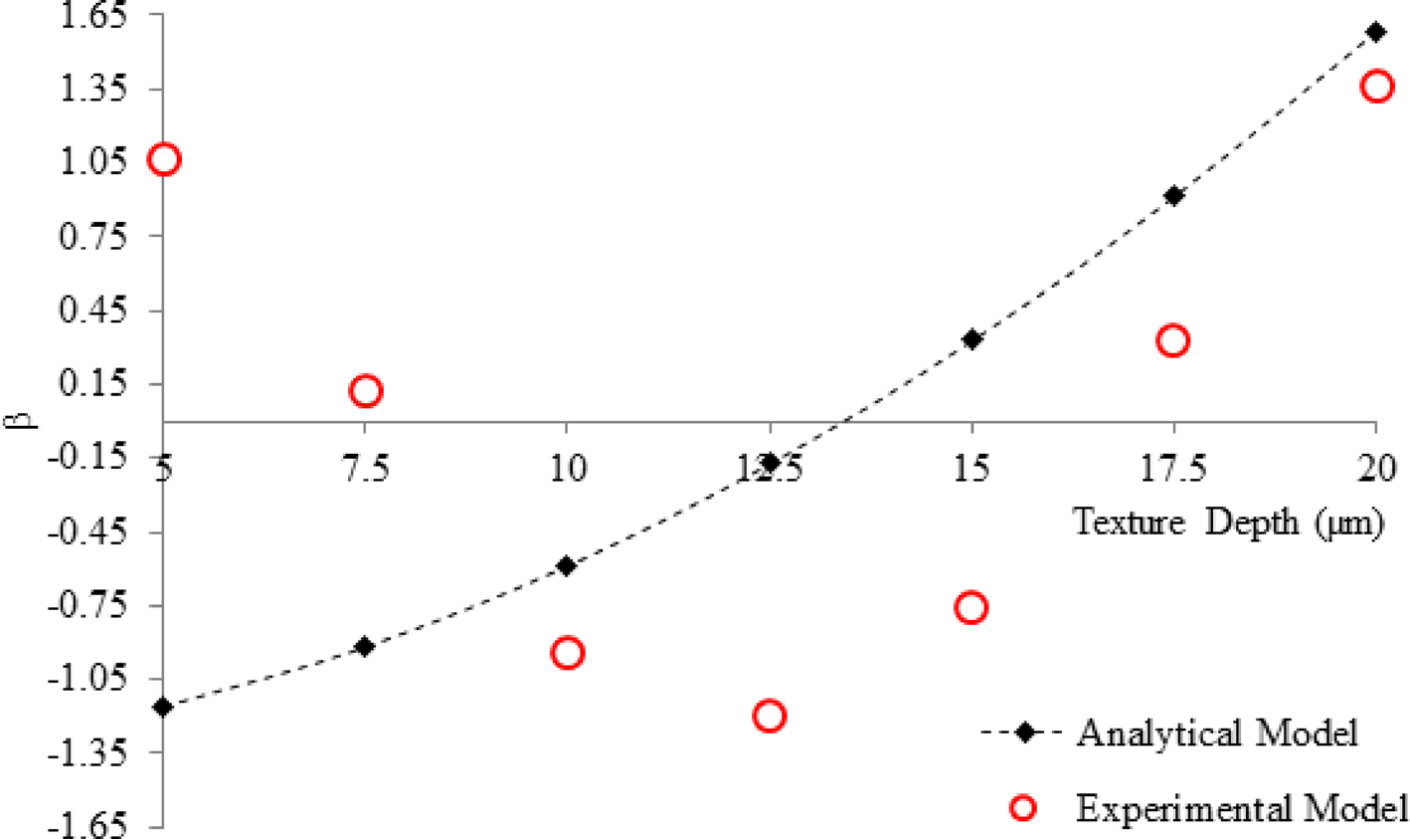
Comparing the standardized friction force for the theoretical and experimental models at various depths in the range from 5 to 20 µm at 2.5 µm intervals

Furthermore, Fig. 16 demonstrates the effect of texture depth. The model developed based on the experimental results provides an optimum depth for friction reduction between 10 and 15 µm. This differs from the theoretical model that exhibits an increase in friction force as the textures deepen and provides no definitive optimum depth. Reasonable trend agreement is attained between the depths of 12.5 µm and 20 µm where both experimental and theoretical friction force increases, suggesting that the model struggles to encapsulate the practical conditions for textures with depths lower than 12.5 µm.

**Fig. 17:**
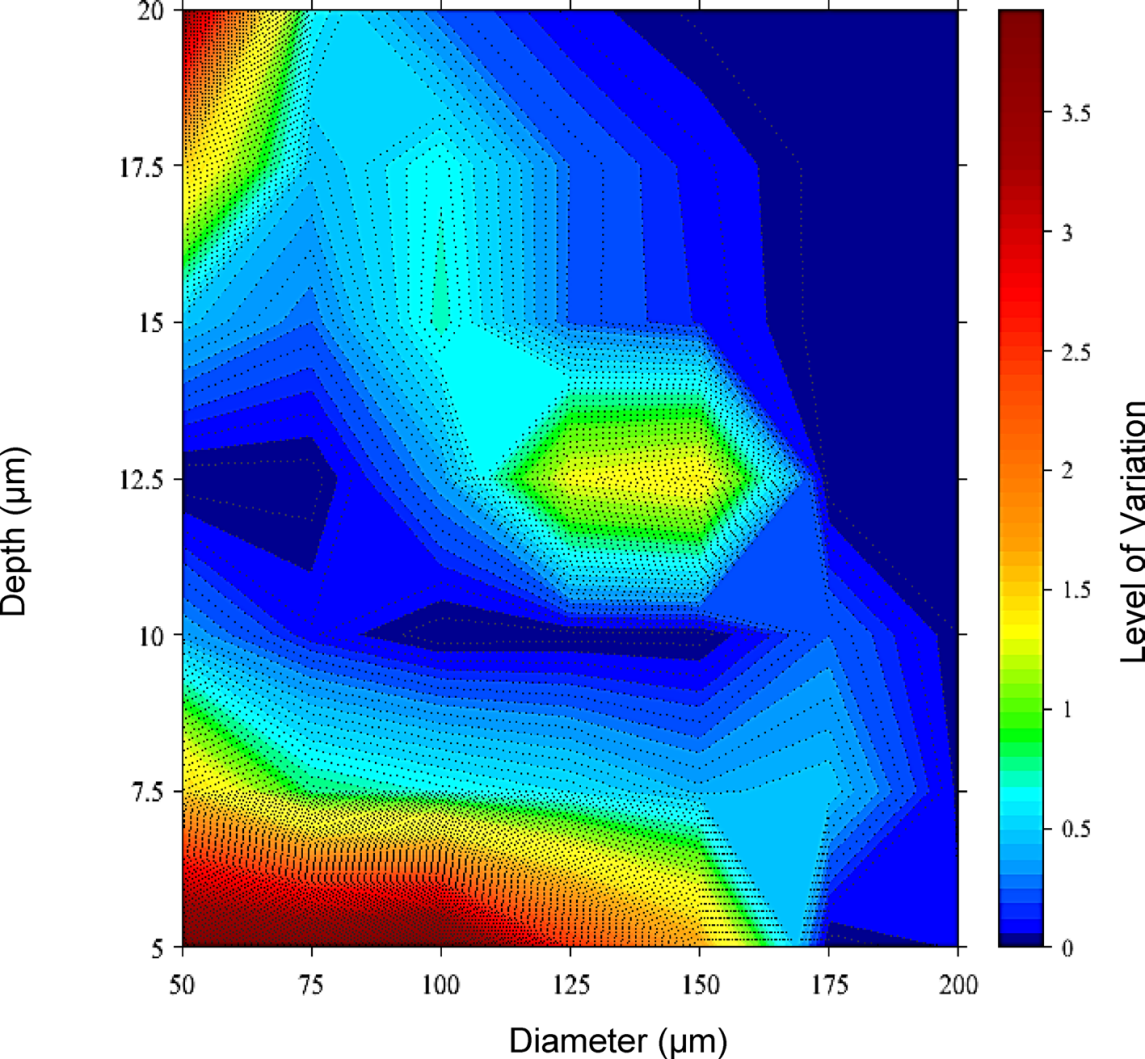
Contour plot and corresponding colour bar expressing the level of variation in β between experimental and theoretical models. The variation is measured by comparing all texture parameter combinations indicated on the depth and diameter axis. The level of variation displayed corresponds to the colour bar on the right of the figure

Figure 17 provides a contour graph illustrating the variation in friction force between the experimental and theoretical models to evaluate where the discrepancies between the experimental and theoretical models reside. It can be observed that the overall model has an acceptable consistency (colours in blue and green), and on some edges, but there are areas where the variation is too high (red regions). The variation in friction force shown in Fig. 17 indicates that the theoretical model's depth and diameter parameter combinations fail to predict the experimental friction force adequately. The level of variance displayed in these instances is attributed to variations in fluid flow throughout the textures. One of the primary assumptions in the theoretical model is that the textures are fully flooded, unless otherwise stated, by the cavitation region around half the circumference of the texture to provide the Reynolds cavitation condition. However, in textures with smaller depths, the total flooding of the pore may be hindered by the physical size of the texture. A mixture of fluid and air may reside within the dimpled pore in the form of a cavitation region at the bottom of the texture, thus reducing the depth over which the fluid operates. Across all the depths employed within the study, Fig. 17 illustrates that the variation in friction force between the experimental and theoretical models is lower for textures of broader diameter than narrower diameter textures. The physical size and volume of the textured pores may affect the fluid's ability to fill the entire texture volume to adhere to the fully flooded assumption, and the notion of a defined optimum aspect ratio is only applicable for textures above a specific diameter.

The existence of a cavitation region at the bottom of the textured pore is assumed to cause discrepancies in the theoretical model when predicting friction force for smaller diameter textures at the extremities of texture depth. The cavitation phenomenon is also relevant in explaining the variation in friction force seen across the texture diameters. However, the cavitation region at the inlet of the texture may vary with a change in diameter. Varying the cavitation region corresponding to a change in the texture diameter would, in turn, increase or decrease the hydrodynamic pressure generated, subsequently causing variations in the friction force. In other words, cavitation occurs when the pressure within the lubricant falls below its vapour pressure, forming vapour bubbles. In smaller-diameter textures with greater depths, the likelihood of cavitation increases due to localised low-pressure zones at the bottom of the pores.

## Conclusions

This study investigated the application of surface textures on PEEK sliding couples, leading to several key findings:

1. Friction Reduction: Textured PEEK surfaces significantly reduce the friction coefficient compared to untextured surfaces, with a 48.7% reduction achieved using a texture combination of 50 µm diameter and 20 µm depth.
2. Theoretical Model Validation: The developed theoretical model accurately predicts friction force, particularly with variations in texture diameter. However, the correlation between empirical and theoretical data is weaker at shallow depths, improving notably at depths above 12.5 µm.
3. Variation Sources: The study identifies cavitation boundary conditions and fully flooded assumptions as primary sources of variation between experimental and theoretical friction data.
4. Tribological Behavior: High-speed friction force data shows good agreement within the hydrodynamic region, despite variations at the start and end of the stroke.

These findings present promising applications for textured PEEK surfaces in biomedical and industrial sectors, emphasizing the importance of future studies to explore different operating conditions like sliding speeds, loads, and temperatures to further validate and extend the theoretical model.

## Data Availability

The datasets generated during and/or analysed during the current study are available from the corresponding author on reasonable request.
